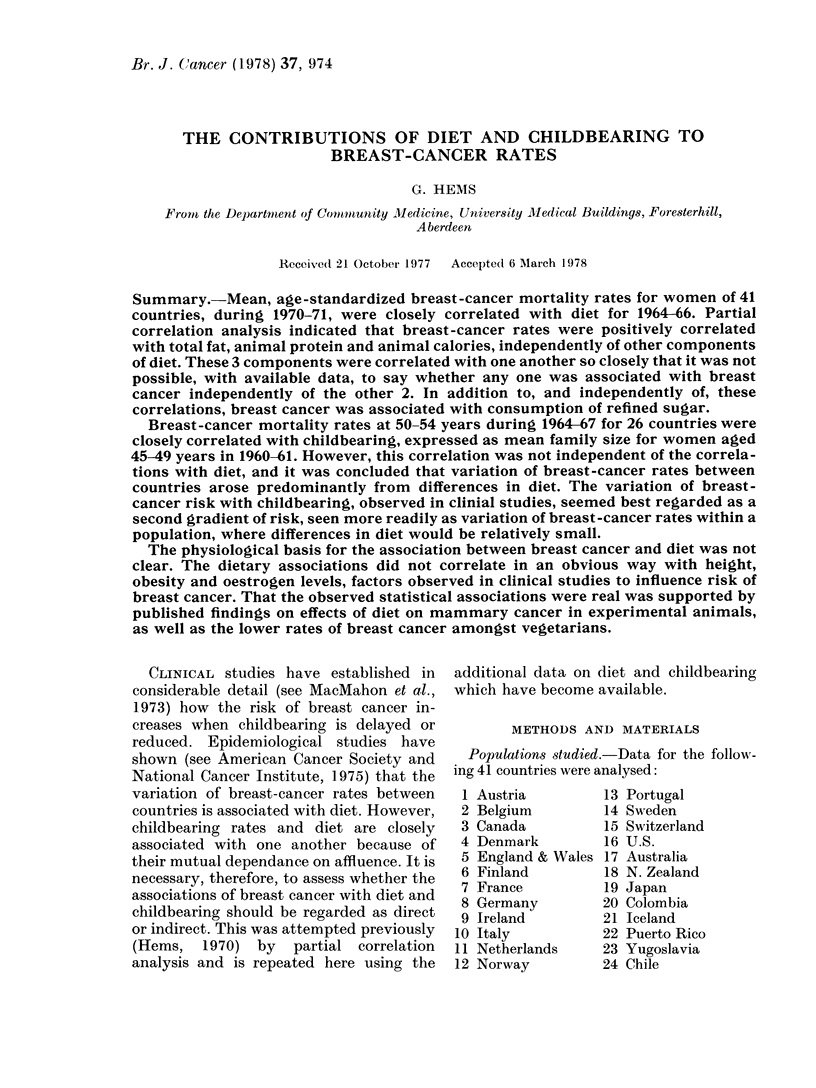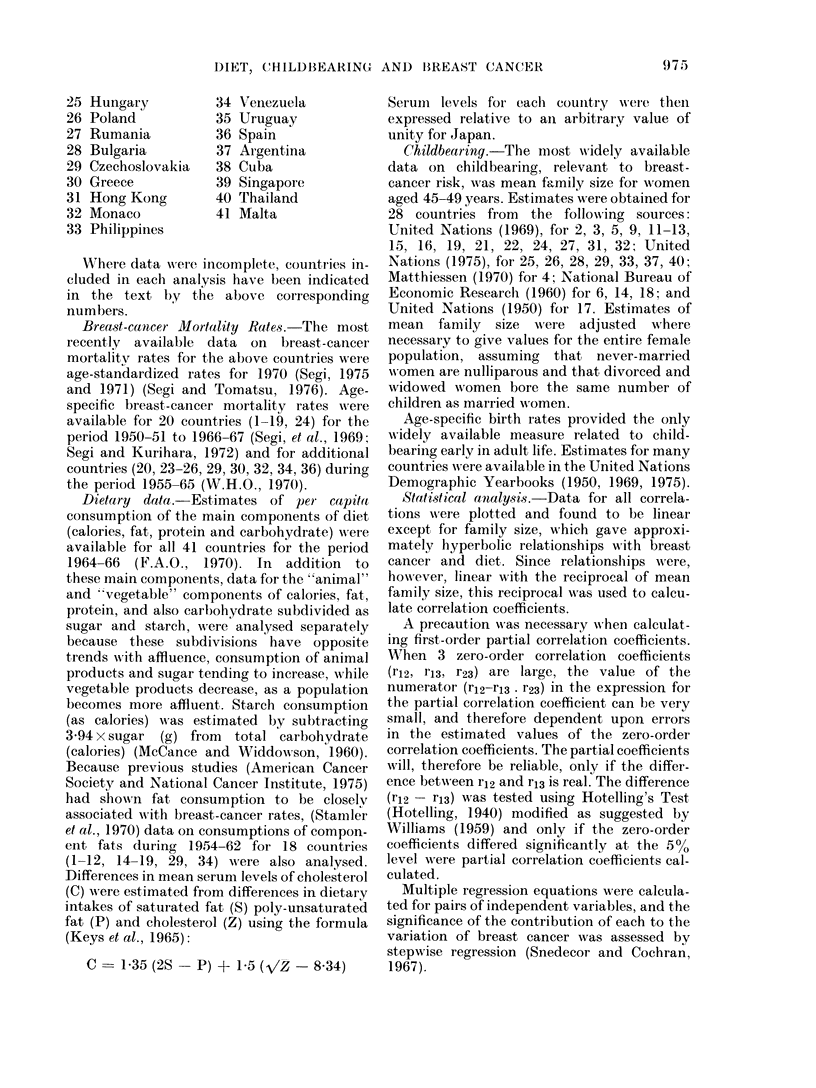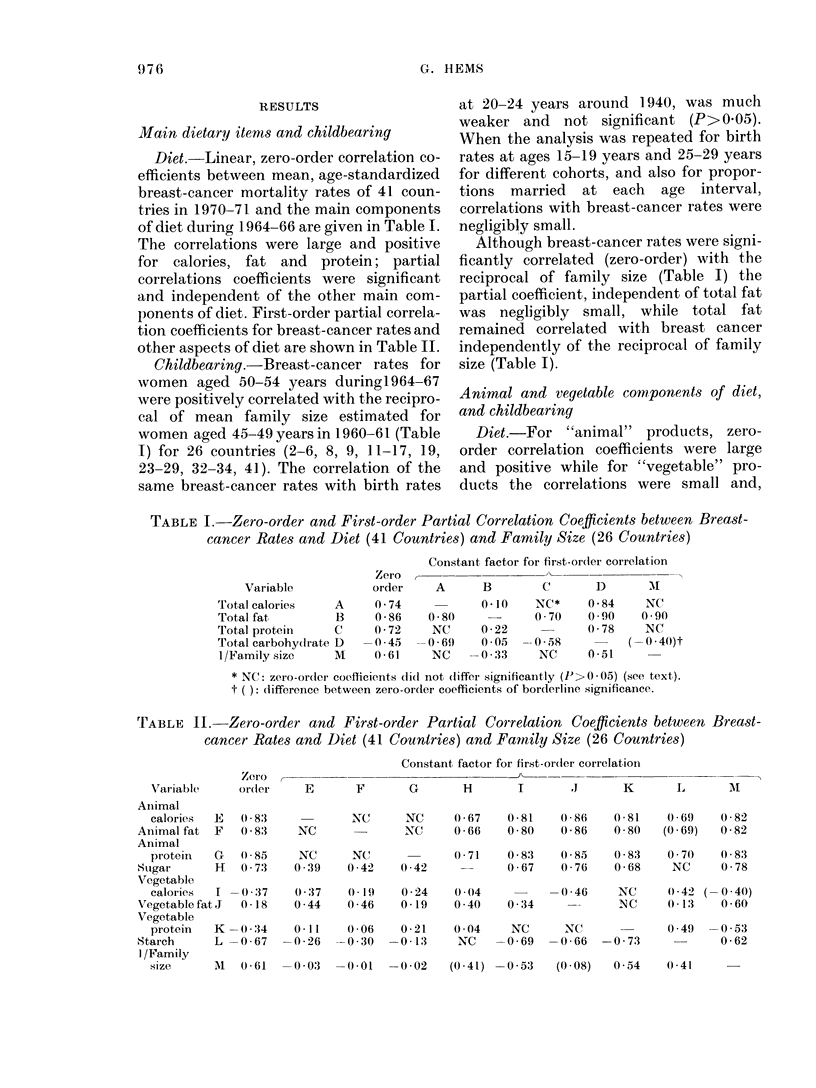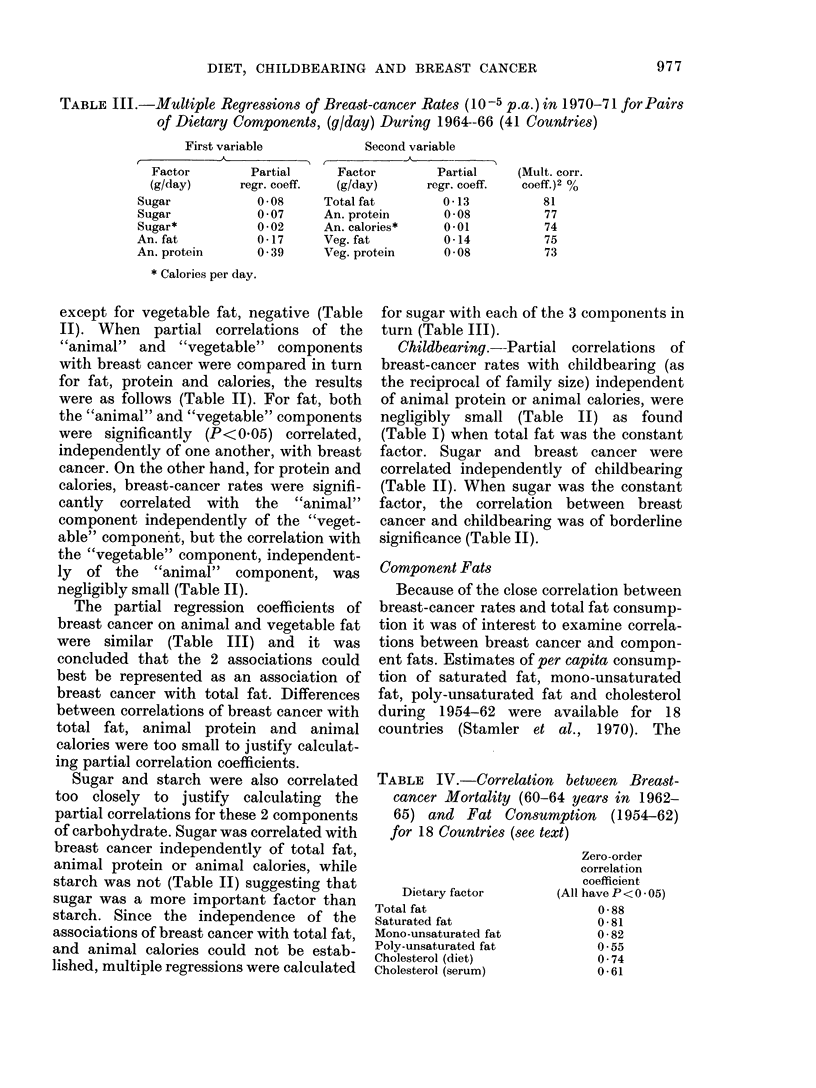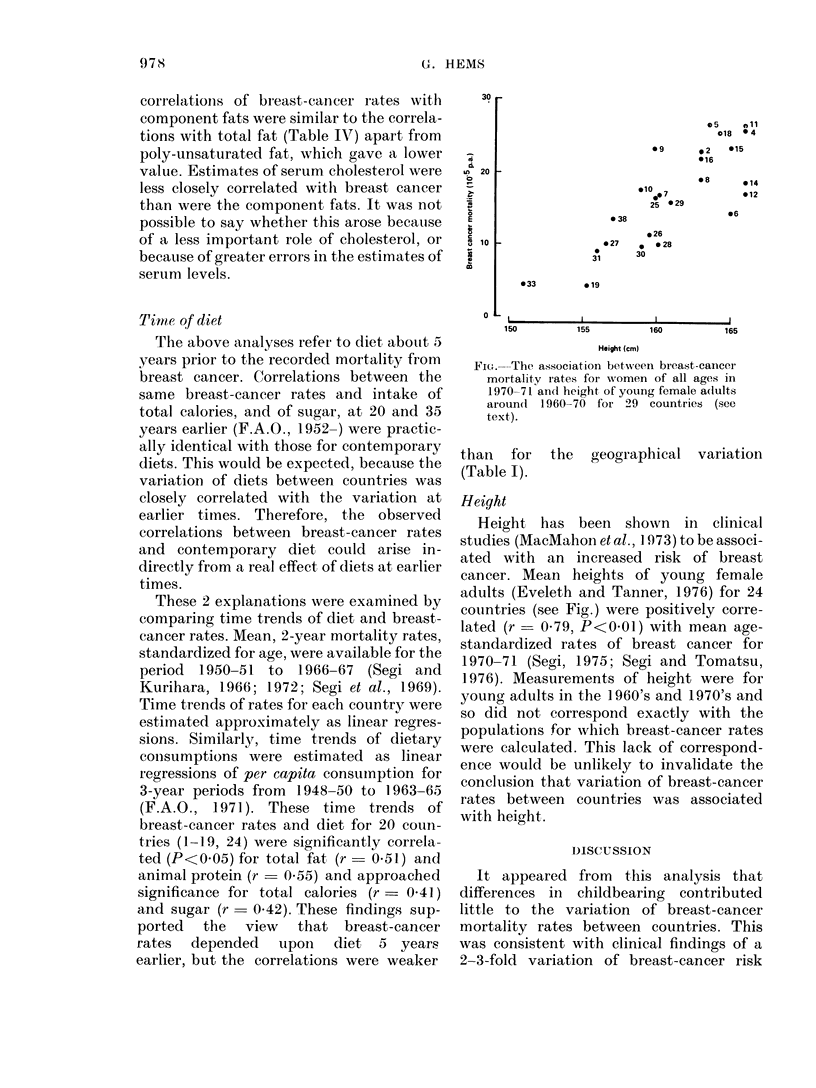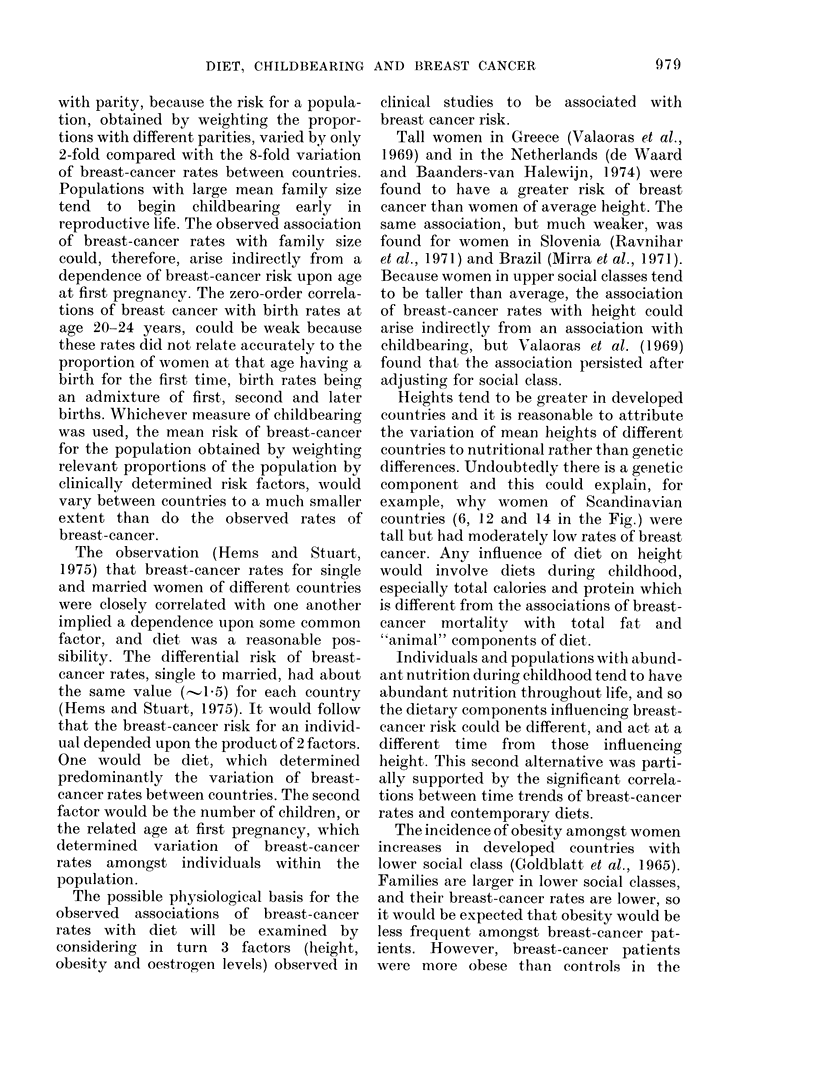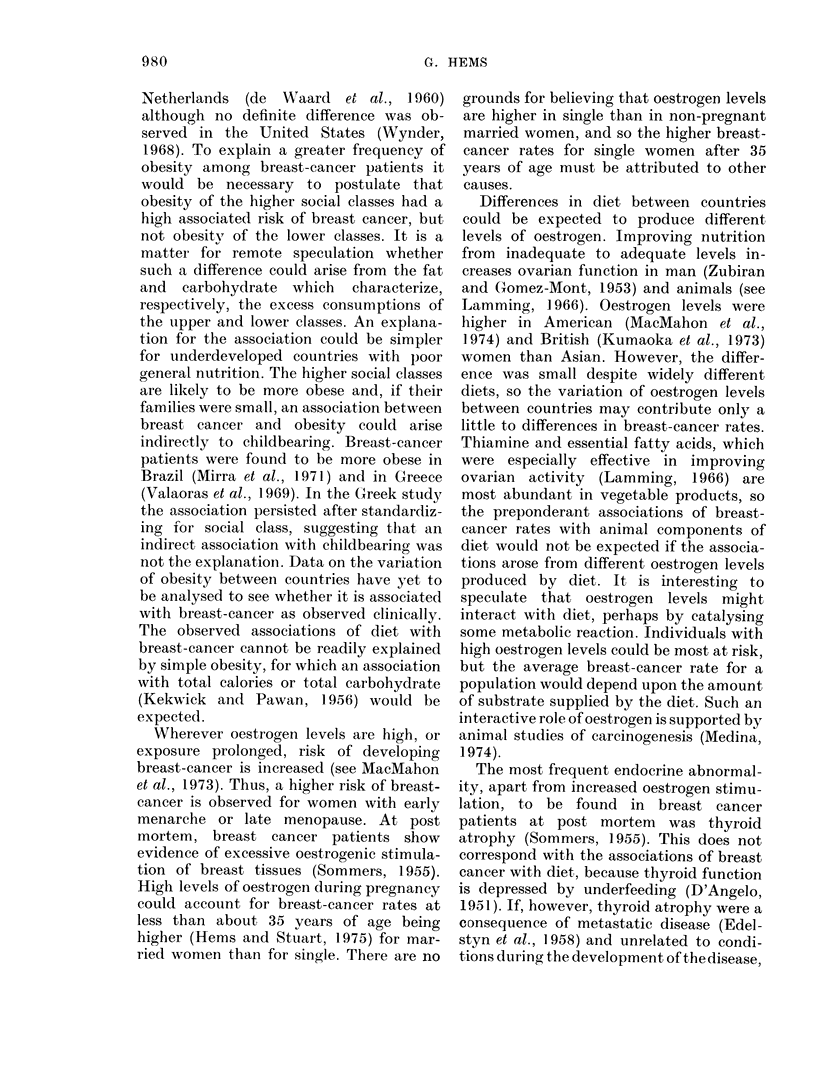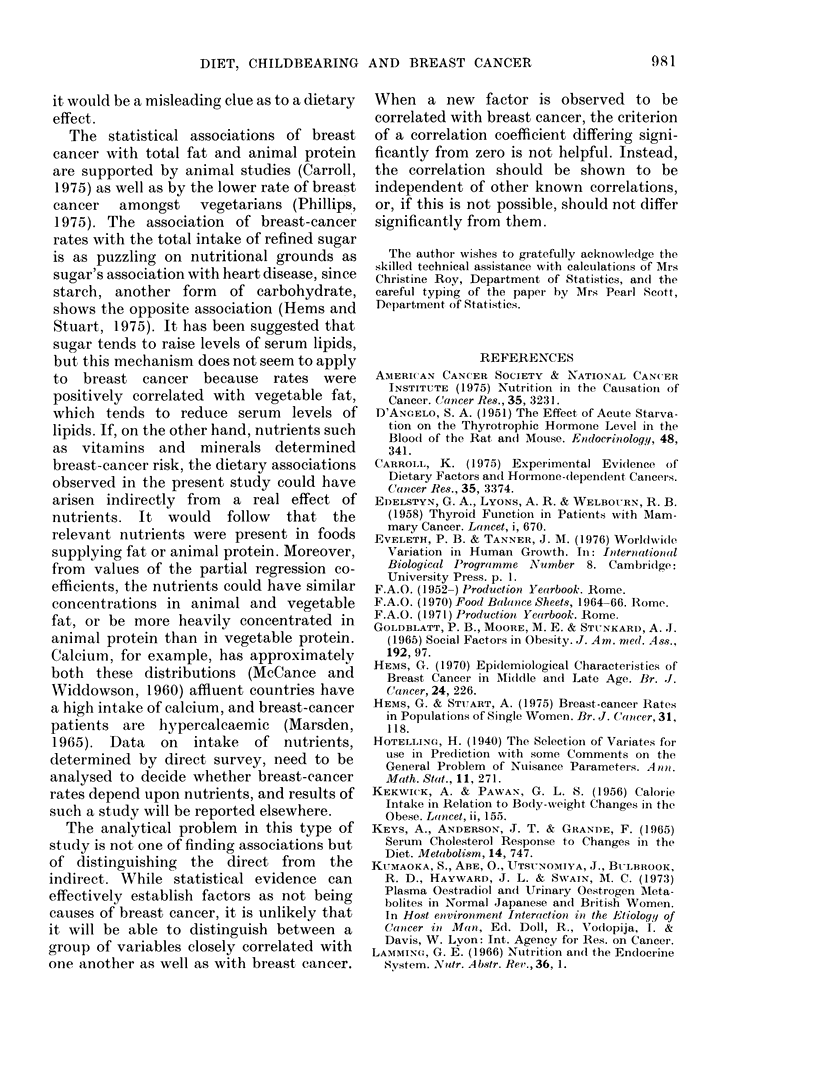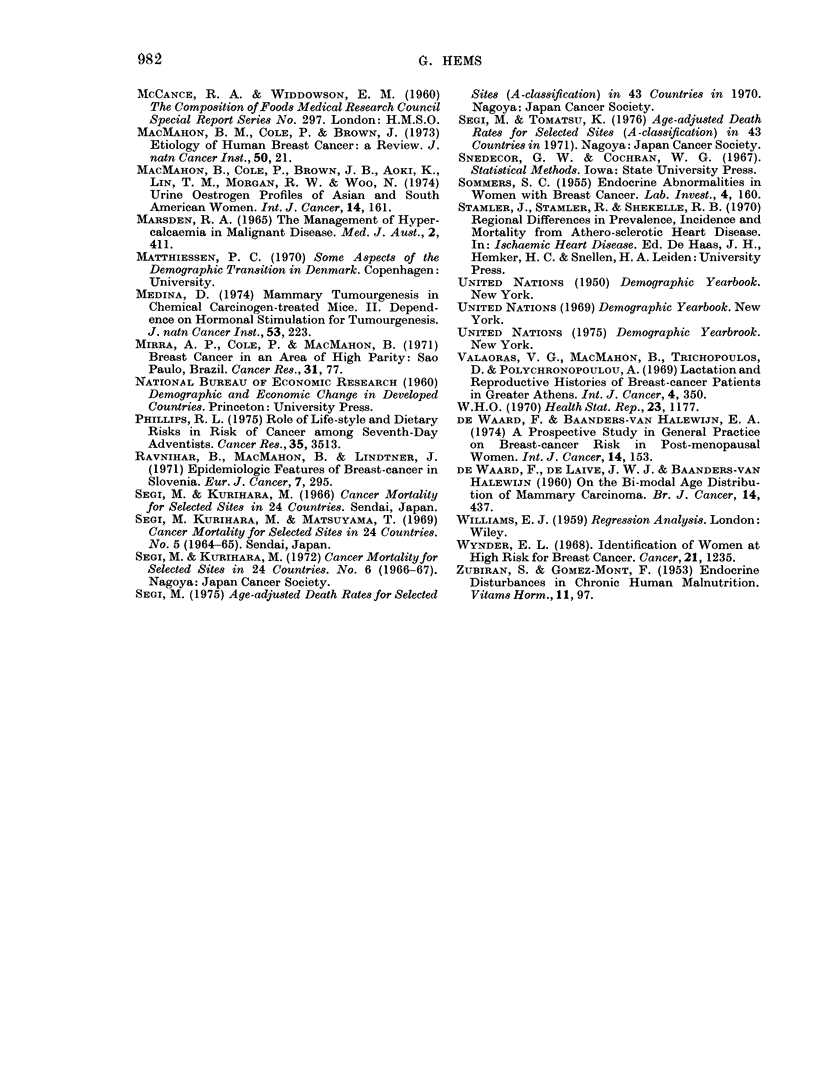# The Contributions of Diet and Childbearing to Breast-cancer Rates

**DOI:** 10.1038/bjc.1978.142

**Published:** 1978-06

**Authors:** G. Hems

## Abstract

Mean, age-standardized breast-cancer mortality rates for women of 41 countries, during 1970-71, were closely correlated with diet for 1964-66. Partial correlation analysis indicated that breast-cancer rates were positively correlated with total fat, animal protein and animal calories, independently of other components of diet. These 3 components were correlated with one another so closely that it was not possible, with available data, to say whether any one was associated with breast cancer independently of the other 2. In addition to, and independently of, these correlations, breast cancer was associated with consumption of refined sugar.

Breast-cancer mortality rates at 50-54 years during 1964-67 for 26 countries were closely correlated with childbearing, expressed as mean family size for women aged 45-49 years in 1960-61. However, this correlation was not independent of the correlations with diet, and it was concluded that variation of breast-cancer rates between countries arose predominantly from differences in diet. The variation of breast-cancer risk with childbearing, observed in clinial studies, seemed best regarded as a second gradient of risk, seen more readily as variation of breast-cancer rates within a population, where differences in diet would be relatively small.

The physiological basis for the association between breast cancer and diet was not clear. The dietary associations did not correlate in an obvious way with height, obesity and oestrogen levels, factors observed in clinical studies to influence risk of breast cancer. That the observed statistical associations were real was supported by published findings on effects of diet on mammary cancer in experimental animals, as well as the lower rates of breast cancer amongst vegetarians.


Br. J. Cancer (1978) 37, 974

### THE CONTRIBUTIONS OF DIET AND CHILDBEARING TO BREAST-CANCER RATES

G. HEMS

*From the Department of Community Medicine, University Medical Buildings, Foresterhill, Aberdeen*

Received 21 October 1977 Accepted 6 March 1978

**Summary.—Mean, age-standardized breast-cancer mortality rates for women of 41 countries, during 1970–71, were closely correlated with diet for 1964–66. Partial correlation analysis indicated that breast-cancer rates were positively correlated with total fat, animal protein and animal calories, independently of other components of diet. These 3 components were correlated with one another so closely that it was not possible, with available data, to say whether any one was associated with breast cancer independently of the other 2. In addition to, and independently of, these correlations, breast cancer was associated with consumption of refined sugar.**

**Breast-cancer mortality rates at 50–54 years during 1964–67 for 26 countries were closely correlated with childbearing, expressed as mean family size for women aged 45–49 years in 1960–61. However, this correlation was not independent of the correlations with diet, and it was concluded that variation of breast-cancer rates between countries arose predominantly from differences in diet. The variation of breast-cancer risk with childbearing, observed in clinial studies, seemed best regarded as a second gradient of risk, seen more readily as variation of breast-cancer rates within a population, where differences in diet would be relatively small.**

**The physiological basis for the association between breast cancer and diet was not clear. The dietary associations did not correlate in an obvious way with height, obesity and oestrogen levels, factors observed in clinical studies to influence risk of breast cancer. That the observed statistical associations were real was supported by published findings on effects of diet on mammary cancer in experimental animals, as well as the lower rates of breast cancer amongst vegetarians.**

CLINICAL studies have established in considerable detail (see MacMahon *et al.*, 1973) how the risk of breast cancer increases when childbearing is delayed or reduced. Epidemiological studies have shown (see American Cancer Society and National Cancer Institute, 1975) that the variation of breast-cancer rates between countries is associated with diet. However, childbearing rates and diet are closely associated with one another because of their mutual dependance on affluence. It is necessary, therefore, to assess whether the associations of breast cancer with diet and childbearing should be regarded as direct or indirect. This was attempted previously (Hems, 1970) by partial correlation analysis and is repeated here using the additional data on diet and childbearing which have become available.

### METHODS AND MATERIALS

*Populations studied.*—Data for the following 41 countries were analysed:

| | |
|---|---|
| 1 Austria | 13 Portugal |
| 2 Belgium | 14 Sweden |
| 3 Canada | 15 Switzerland |
| 4 Denmark | 16 U.S. |
| 5 England & Wales | 17 Australia |
| 6 Finland | 18 N. Zealand |
| 7 France | 19 Japan |
| 8 Germany | 20 Colombia |
| 9 Ireland | 21 Iceland |
| 10 Italy | 22 Puerto Rico |
| 11 Netherlands | 23 Yugoslavia |
| 12 Norway | 24 Chile |

25 Hungary

26 Poland

27 Rumania

28 Bulgaria

29 Czechoslovakia

30 Greece

31 Hong Kong

32 Monaco

33 Philippines

34 Venezuela

35 Uruguay

36 Spain

37 Argentina

38 Cuba

39 Singapore

40 Thailand

41 Malta

Where data were incomplete, countries included in each analysis have been indicated in the text by the above corresponding numbers.

*Breast-cancer Mortality Rates*.—The most recently available data on breast-cancer mortality rates for the above countries were age-standardized rates for 1970 (Segi, 1975 and 1971) (Segi and Tomatsu, 1976). Age-specific breast-cancer mortality rates were available for 20 countries (1–19, 24) for the period 1950–51 to 1966–67 (Segi, *et al.*, 1969; Segi and Kurihara, 1972) and for additional countries (20, 23–26, 29, 30, 32, 34, 36) during the period 1955–65 (W.H.O., 1970).

*Dietary data*.—Estimates of *per capita* consumption of the main components of diet (calories, fat, protein and carbohydrate) were available for all 41 countries for the period 1964–66 (F.A.O., 1970). In addition to these main components, data for the "animal" and "vegetable" components of calories, fat, protein, and also carbohydrate subdivided as sugar and starch, were analysed separately because these subdivisions have opposite trends with affluence, consumption of animal products and sugar tending to increase, while vegetable products decrease, as a population becomes more affluent. Starch consumption (as calories) was estimated by subtracting $3{\cdot}94 \times$ sugar (g) from total carbohydrate (calories) (McCance and Widdowson, 1960). Because previous studies (American Cancer Society and National Cancer Institute, 1975) had shown fat consumption to be closely associated with breast-cancer rates, (Stamler *et al.*, 1970) data on consumptions of component fats during 1954–62 for 18 countries (1–12, 14–19, 29, 34) were also analysed. Differences in mean serum levels of cholesterol (C) were estimated from differences in dietary intakes of saturated fat (S) poly-unsaturated fat (P) and cholesterol (Z) using the formula (Keys *et al.*, 1965):

$$C = 1{\cdot}35\,(2S - P) + 1{\cdot}5\,(\sqrt{Z} - 8{\cdot}34)$$

Serum levels for each country were then expressed relative to an arbitrary value of unity for Japan.

*Childbearing*.—The most widely available data on childbearing, relevant to breast-cancer risk, was mean family size for women aged 45–49 years. Estimates were obtained for 28 countries from the following sources: United Nations (1969), for 2, 3, 5, 9, 11–13, 15, 16, 19, 21, 22, 24, 27, 31, 32; United Nations (1975), for 25, 26, 28, 29, 33, 37, 40; Matthiessen (1970) for 4; National Bureau of Economic Research (1960) for 6, 14, 18; and United Nations (1950) for 17. Estimates of mean family size were adjusted where necessary to give values for the entire female population, assuming that never-married women are nulliparous and that divorced and widowed women bore the same number of children as married women.

Age-specific birth rates provided the only widely available measure related to childbearing early in adult life. Estimates for many countries were available in the United Nations Demographic Yearbooks (1950, 1969, 1975).

*Statistical analysis*.—Data for all correlations were plotted and found to be linear except for family size, which gave approximately hyperbolic relationships with breast cancer and diet. Since relationships were, however, linear with the reciprocal of mean family size, this reciprocal was used to calculate correlation coefficients.

A precaution was necessary when calculating first-order partial correlation coefficients. When 3 zero-order correlation coefficients ($r_{12}$, $r_{13}$, $r_{23}$) are large, the value of the numerator ($r_{12} - r_{13} \cdot r_{23}$) in the expression for the partial correlation coefficient can be very small, and therefore dependent upon errors in the estimated values of the zero-order correlation coefficients. The partial coefficients will, therefore be reliable, only if the difference between $r_{12}$ and $r_{13}$ is real. The difference ($r_{12} - r_{13}$) was tested using Hotelling's Test (Hotelling, 1940) modified as suggested by Williams (1959) and only if the zero-order coefficients differed significantly at the 5% level were partial correlation coefficients calculated.

Multiple regression equations were calculated for pairs of independent variables, and the significance of the contribution of each to the variation of breast cancer was assessed by stepwise regression (Snedecor and Cochran, 1967).

### RESULTS

### *Main dietary items and childbearing*

*Diet.*—Linear, zero-order correlation coefficients between mean, age-standardized breast-cancer mortality rates of 41 countries in 1970–71 and the main components of diet during 1964–66 are given in Table I. The correlations were large and positive for calories, fat and protein; partial correlations coefficients were significant and independent of the other main components of diet. First-order partial correlation coefficients for breast-cancer rates and other aspects of diet are shown in Table II.

*Childbearing.*—Breast-cancer rates for women aged 50–54 years during1964–67 were positively correlated with the reciprocal of mean family size estimated for women aged 45–49 years in 1960–61 (Table I) for 26 countries (2–6, 8, 9, 11–17, 19, 23-29, 32–34, 41). The correlation of the same breast-cancer rates with birth rates at 20–24 years around 1940, was much weaker and not significant ($P>0·05$). When the analysis was repeated for birth rates at ages 15–19 years and 25–29 years for different cohorts, and also for proportions married at each age interval, correlations with breast-cancer rates were negligibly small.

Although breast-cancer rates were significantly correlated (zero-order) with the reciprocal of family size (Table I) the partial coefficient, independent of total fat was negligibly small, while total fat remained correlated with breast cancer independently of the reciprocal of family size (Table I).

### *Animal and vegetable components of diet, and childbearing*

*Diet.*—For "animal" products, zero-order correlation coefficients were large and positive while for "vegetable" products the correlations were small and,

TABLE I.—*Zero-order and First-order Partial Correlation Coefficients between Breast-cancer Rates and Diet* (41 *Countries*) *and Family Size* (26 *Countries*)

| Variable | | Zero order | Constant factor for first-order correlation A | B | C | D | M |
|---|---|---|---|---|---|---|---|
| Total calories | A | 0·74 | — | 0·10 | NC* | 0·84 | NC |
| Total fat | B | 0·86 | 0·80 | — | 0·70 | 0·90 | 0·90 |
| Total protein | C | 0·72 | NC | 0·22 | — | 0·78 | NC |
| Total carbohydrate | D | −0·45 | −0·69 | 0·05 | −0·58 | — | (−0·40)† |
| 1/Family size | M | 0·61 | NC | −0·33 | NC | 0·51 | — |

* NC: zero-order coefficients did not differ significantly ($P>0·05$) (see text).
† ( ): difference between zero-order coefficients of borderline significance.

TABLE II.—*Zero-order and First-order Partial Correlation Coefficients between Breast-cancer Rates and Diet* (41 *Countries*) *and Family Size* (26 *Countries*)

| Variable | | Zero order | Constant factor for first-order correlation E | F | G | H | I | J | K | L | M |
|---|---|---|---|---|---|---|---|---|---|---|---|
| Animal calories | E | 0·83 | — | NC | NC | 0·67 | 0·81 | 0·86 | 0·81 | 0·69 | 0·82 |
| Animal fat | F | 0·83 | NC | — | NC | 0·66 | 0·80 | 0·86 | 0·80 | (0·69) | 0·82 |
| Animal protein | G | 0·85 | NC | NC | — | 0·71 | 0·83 | 0·85 | 0·83 | 0·70 | 0·83 |
| Sugar | H | 0·73 | 0·39 | 0·42 | 0·42 | — | 0·67 | 0·76 | 0·68 | NC | 0·78 |
| Vegetable calories | I | −0·37 | 0·37 | 0·19 | 0·24 | 0·04 | — | −0·46 | NC | 0·42 | (−0·40) |
| Vegetable fat | J | 0·18 | 0·44 | 0·46 | 0·19 | 0·40 | 0·34 | — | NC | 0·13 | 0·60 |
| Vegetable protein | K | −0·34 | 0·11 | 0·06 | 0·21 | 0·04 | NC | NC | — | 0·49 | −0·53 |
| Starch | L | −0·67 | −0·26 | −0·30 | −0·13 | NC | −0·69 | −0·66 | −0·73 | — | 0·62 |
| 1/Family size | M | 0·61 | −0·03 | −0·01 | −0·02 | (0·41) | −0·53 | (0·08) | 0·54 | 0·41 | — |

TABLE III.—*Multiple Regressions of Breast-cancer Rates* ($10^{-5}$ *p.a.*) *in* 1970–71 *for Pairs of Dietary Components,* (*g/day*) *During* 1964–66 (41 *Countries*)

| First variable | | Second variable | | |
|---|---|---|---|---|
| Factor (g/day) | Partial regr. coeff. | Factor (g/day) | Partial regr. coeff. | (Mult. corr. coeff.)$^2$ % |
| Sugar | 0·08 | Total fat | 0·13 | 81 |
| Sugar | 0·07 | An. protein | 0·08 | 77 |
| Sugar* | 0·02 | An. calories* | 0·01 | 74 |
| An. fat | 0·17 | Veg. fat | 0·14 | 75 |
| An. protein | 0·39 | Veg. protein | 0·08 | 73 |

* Calories per day.

except for vegetable fat, negative (Table II). When partial correlations of the "animal" and "vegetable" components with breast cancer were compared in turn for fat, protein and calories, the results were as follows (Table II). For fat, both the "animal" and "vegetable" components were significantly ($P<0{\cdot}05$) correlated, independently of one another, with breast cancer. On the other hand, for protein and calories, breast-cancer rates were significantly correlated with the "animal" component independently of the "vegetable" component, but the correlation with the "vegetable" component, independently of the "animal" component, was negligibly small (Table II).

The partial regression coefficients of breast cancer on animal and vegetable fat were similar (Table III) and it was concluded that the 2 associations could best be represented as an association of breast cancer with total fat. Differences between correlations of breast cancer with total fat, animal protein and animal calories were too small to justify calculating partial correlation coefficients.

Sugar and starch were also correlated too closely to justify calculating the partial correlations for these 2 components of carbohydrate. Sugar was correlated with breast cancer independently of total fat, animal protein or animal calories, while starch was not (Table II) suggesting that sugar was a more important factor than starch. Since the independence of the associations of breast cancer with total fat, and animal calories could not be established, multiple regressions were calculated for sugar with each of the 3 components in turn (Table III).

*Childbearing.*—Partial correlations of breast-cancer rates with childbearing (as the reciprocal of family size) independent of animal protein or animal calories, were negligibly small (Table II) as found (Table I) when total fat was the constant factor. Sugar and breast cancer were correlated independently of childbearing (Table II). When sugar was the constant factor, the correlation between breast cancer and childbearing was of borderline significance (Table II).

*Component Fats*

Because of the close correlation between breast-cancer rates and total fat consumption it was of interest to examine correlations between breast cancer and component fats. Estimates of *per capita* consumption of saturated fat, mono-unsaturated fat, poly-unsaturated fat and cholesterol during 1954–62 were available for 18 countries (Stamler *et al.*, 1970). The

TABLE IV.—*Correlation between Breast-cancer Mortality* (60–64 *years in* 1962–65) *and Fat Consumption* (1954–62) *for* 18 *Countries* (*see text*)

| Dietary factor | Zero-order correlation coefficient (All have $P<0{\cdot}05$) |
|---|---|
| Total fat | 0·88 |
| Saturated fat | 0·81 |
| Mono-unsaturated fat | 0·82 |
| Poly-unsaturated fat | 0·55 |
| Cholesterol (diet) | 0·74 |
| Cholesterol (serum) | 0·61 |

correlations of breast-cancer rates with component fats were similar to the correlations with total fat (Table IV) apart from poly-unsaturated fat, which gave a lower value. Estimates of serum cholesterol were less closely correlated with breast cancer than were the component fats. It was not possible to say whether this arose because of a less important role of cholesterol, or because of greater errors in the estimates of serum levels.

*Time of diet*

The above analyses refer to diet about 5 years prior to the recorded mortality from breast cancer. Correlations between the same breast-cancer rates and intake of total calories, and of sugar, at 20 and 35 years earlier (F.A.O., 1952–) were practically identical with those for contemporary diets. This would be expected, because the variation of diets between countries was closely correlated with the variation at earlier times. Therefore, the observed correlations between breast-cancer rates and contemporary diet could arise indirectly from a real effect of diets at earlier times.

These 2 explanations were examined by comparing time trends of diet and breast-cancer rates. Mean, 2-year mortality rates, standardized for age, were available for the period 1950–51 to 1966–67 (Segi and Kurihara, 1966; 1972; Segi *et al.*, 1969). Time trends of rates for each country were estimated approximately as linear regressions. Similarly, time trends of dietary consumptions were estimated as linear regressions of *per capita* consumption for 3-year periods from 1948–50 to 1963–65 (F.A.O., 1971). These time trends of breast-cancer rates and diet for 20 countries (1–19, 24) were significantly correlated ($P<0{\cdot}05$) for total fat ($r = 0{\cdot}51$) and animal protein ($r = 0{\cdot}55$) and approached significance for total calories ($r = 0{\cdot}41$) and sugar ($r = 0{\cdot}42$). These findings supported the view that breast-cancer rates depended upon diet 5 years earlier, but the correlations were weaker

FIG.—The association between breast-cancer mortality rates for women of all ages in 1970–71 and height of young female adults around 1960–70 for 29 countries (see text).

than for the geographical variation (Table I).

*Height*

Height has been shown in clinical studies (MacMahon *et al.*, 1973) to be associated with an increased risk of breast cancer. Mean heights of young female adults (Eveleth and Tanner, 1976) for 24 countries (see Fig.) were positively correlated ($r = 0{\cdot}79$, $P<0{\cdot}01$) with mean age-standardized rates of breast cancer for 1970–71 (Segi, 1975; Segi and Tomatsu, 1976). Measurements of height were for young adults in the 1960's and 1970's and so did not correspond exactly with the populations for which breast-cancer rates were calculated. This lack of correspondence would be unlikely to invalidate the conclusion that variation of breast-cancer rates between countries was associated with height.

### DISCUSSION

It appeared from this analysis that differences in childbearing contributed little to the variation of breast-cancer mortality rates between countries. This was consistent with clinical findings of a 2–3-fold variation of breast-cancer risk with parity, because the risk for a population, obtained by weighting the proportions with different parities, varied by only 2-fold compared with the 8-fold variation of breast-cancer rates between countries. Populations with large mean family size tend to begin childbearing early in reproductive life. The observed association of breast-cancer rates with family size could, therefore, arise indirectly from a dependence of breast-cancer risk upon age at first pregnancy. The zero-order correlations of breast cancer with birth rates at age 20–24 years, could be weak because these rates did not relate accurately to the proportion of women at that age having a birth for the first time, birth rates being an admixture of first, second and later births. Whichever measure of childbearing was used, the mean risk of breast-cancer for the population obtained by weighting relevant proportions of the population by clinically determined risk factors, would vary between countries to a much smaller extent than do the observed rates of breast-cancer.

The observation (Hems and Stuart, 1975) that breast-cancer rates for single and married women of different countries were closely correlated with one another implied a dependence upon some common factor, and diet was a reasonable possibility. The differential risk of breast-cancer rates, single to married, had about the same value ($\sim$1·5) for each country (Hems and Stuart, 1975). It would follow that the breast-cancer risk for an individual depended upon the product of 2 factors. One would be diet, which determined predominantly the variation of breast-cancer rates between countries. The second factor would be the number of children, or the related age at first pregnancy, which determined variation of breast-cancer rates amongst individuals within the population.

The possible physiological basis for the observed associations of breast-cancer rates with diet will be examined by considering in turn 3 factors (height, obesity and oestrogen levels) observed in clinical studies to be associated with breast cancer risk.

Tall women in Greece (Valaoras *et al.*, 1969) and in the Netherlands (de Waard and Baanders-van Halewijn, 1974) were found to have a greater risk of breast cancer than women of average height. The same association, but much weaker, was found for women in Slovenia (Ravnihar *et al.*, 1971) and Brazil (Mirra *et al.*, 1971). Because women in upper social classes tend to be taller than average, the association of breast-cancer rates with height could arise indirectly from an association with childbearing, but Valaoras *et al.* (1969) found that the association persisted after adjusting for social class.

Heights tend to be greater in developed countries and it is reasonable to attribute the variation of mean heights of different countries to nutritional rather than genetic differences. Undoubtedly there is a genetic component and this could explain, for example, why women of Scandinavian countries (6, 12 and 14 in the Fig.) were tall but had moderately low rates of breast cancer. Any influence of diet on height would involve diets during childhood, especially total calories and protein which is different from the associations of breast-cancer mortality with total fat and "animal" components of diet.

Individuals and populations with abundant nutrition during childhood tend to have abundant nutrition throughout life, and so the dietary components influencing breast-cancer risk could be different, and act at a different time from those influencing height. This second alternative was partially supported by the significant correlations between time trends of breast-cancer rates and contemporary diets.

The incidence of obesity amongst women increases in developed countries with lower social class (Goldblatt *et al.*, 1965). Families are larger in lower social classes, and their breast-cancer rates are lower, so it would be expected that obesity would be less frequent amongst breast-cancer patients. However, breast-cancer patients were more obese than controls in the

Netherlands (de Waard *et al.*, 1960) although no definite difference was observed in the United States (Wynder, 1968). To explain a greater frequency of obesity among breast-cancer patients it would be necessary to postulate that obesity of the higher social classes had a high associated risk of breast cancer, but not obesity of the lower classes. It is a matter for remote speculation whether such a difference could arise from the fat and carbohydrate which characterize, respectively, the excess consumptions of the upper and lower classes. An explanation for the association could be simpler for underdeveloped countries with poor general nutrition. The higher social classes are likely to be more obese and, if their families were small, an association between breast cancer and obesity could arise indirectly to childbearing. Breast-cancer patients were found to be more obese in Brazil (Mirra *et al.*, 1971) and in Greece (Valaoras *et al.*, 1969). In the Greek study the association persisted after standardizing for social class, suggesting that an indirect association with childbearing was not the explanation. Data on the variation of obesity between countries have yet to be analysed to see whether it is associated with breast-cancer as observed clinically. The observed associations of diet with breast-cancer cannot be readily explained by simple obesity, for which an association with total calories or total carbohydrate (Kekwick and Pawan, 1956) would be expected.

Wherever oestrogen levels are high, or exposure prolonged, risk of developing breast-cancer is increased (see MacMahon *et al.*, 1973). Thus, a higher risk of breast-cancer is observed for women with early menarche or late menopause. At post mortem, breast cancer patients show evidence of excessive oestrogenic stimulation of breast tissues (Sommers, 1955). High levels of oestrogen during pregnancy could account for breast-cancer rates at less than about 35 years of age being higher (Hems and Stuart, 1975) for married women than for single. There are no grounds for believing that oestrogen levels are higher in single than in non-pregnant married women, and so the higher breast-cancer rates for single women after 35 years of age must be attributed to other causes.

Differences in diet between countries could be expected to produce different levels of oestrogen. Improving nutrition from inadequate to adequate levels increases ovarian function in man (Zubiran and Gomez-Mont, 1953) and animals (see Lamming, 1966). Oestrogen levels were higher in American (MacMahon *et al.*, 1974) and British (Kumaoka *et al.*, 1973) women than Asian. However, the difference was small despite widely different diets, so the variation of oestrogen levels between countries may contribute only a little to differences in breast-cancer rates. Thiamine and essential fatty acids, which were especially effective in improving ovarian activity (Lamming, 1966) are most abundant in vegetable products, so the preponderant associations of breast-cancer rates with animal components of diet would not be expected if the associations arose from different oestrogen levels produced by diet. It is interesting to speculate that oestrogen levels might interact with diet, perhaps by catalysing some metabolic reaction. Individuals with high oestrogen levels could be most at risk, but the average breast-cancer rate for a population would depend upon the amount of substrate supplied by the diet. Such an interactive role of oestrogen is supported by animal studies of carcinogenesis (Medina, 1974).

The most frequent endocrine abnormality, apart from increased oestrogen stimulation, to be found in breast cancer patients at post mortem was thyroid atrophy (Sommers, 1955). This does not correspond with the associations of breast cancer with diet, because thyroid function is depressed by underfeeding (D'Angelo, 1951). If, however, thyroid atrophy were a consequence of metastatic disease (Edelstyn *et al.*, 1958) and unrelated to conditions during the development of the disease, it would be a misleading clue as to a dietary effect.

The statistical associations of breast cancer with total fat and animal protein are supported by animal studies (Carroll, 1975) as well as by the lower rate of breast cancer amongst vegetarians (Phillips, 1975). The association of breast-cancer rates with the total intake of refined sugar is as puzzling on nutritional grounds as sugar's association with heart disease, since starch, another form of carbohydrate, shows the opposite association (Hems and Stuart, 1975). It has been suggested that sugar tends to raise levels of serum lipids, but this mechanism does not seem to apply to breast cancer because rates were positively correlated with vegetable fat, which tends to reduce serum levels of lipids. If, on the other hand, nutrients such as vitamins and minerals determined breast-cancer risk, the dietary associations observed in the present study could have arisen indirectly from a real effect of nutrients. It would follow that the relevant nutrients were present in foods supplying fat or animal protein. Moreover, from values of the partial regression coefficients, the nutrients could have similar concentrations in animal and vegetable fat, or be more heavily concentrated in animal protein than in vegetable protein. Calcium, for example, has approximately both these distributions (McCance and Widdowson, 1960) affluent countries have a high intake of calcium, and breast-cancer patients are hypercalcaemic (Marsden, 1965). Data on intake of nutrients, determined by direct survey, need to be analysed to decide whether breast-cancer rates depend upon nutrients, and results of such a study will be reported elsewhere.

The analytical problem in this type of study is not one of finding associations but of distinguishing the direct from the indirect. While statistical evidence can effectively establish factors as not being causes of breast cancer, it is unlikely that it will be able to distinguish between a group of variables closely correlated with one another as well as with breast cancer. When a new factor is observed to be correlated with breast cancer, the criterion of a correlation coefficient differing significantly from zero is not helpful. Instead, the correlation should be shown to be independent of other known correlations, or, if this is not possible, should not differ significantly from them.

The author wishes to gratefully acknowledge the skilled technical assistance with calculations of Mrs Christine Roy, Department of Statistics, and the careful typing of the paper by Mrs Pearl Scott, Department of Statistics.

### REFERENCES

American Cancer Society & National Cancer Institute (1975) Nutrition in the Causation of Cancer. *Cancer Res.*, **35**, 3231.

D'Angelo, S. A. (1951) The Effect of Acute Starvation on the Thyrotrophic Hormone Level in the Blood of the Rat and Mouse. *Endocrinology*, **48**, 341.

Carroll, K. (1975) Experimental Evidence of Dietary Factors and Hormone-dependent Cancers. *Cancer Res.*, **35**, 3374.

Edelstyn, G. A., Lyons, A. R. & Welbourn, R. B. (1958) Thyroid Function in Patients with Mammary Cancer. *Lancet*, i, 670.

Eveleth, P. B. & Tanner, J. M. (1976) Worldwide Variation in Human Growth. In: *International Biological Programme Number* 8. Cambridge: University Press. p. 1.

F.A.O. (1952–) *Production Yearbook*. Rome.

F.A.O. (1970) *Food Balance Sheets*, 1964–66. Rome.

F.A.O. (1971) *Production Yearbook*. Rome.

Goldblatt, P. B., Moore, M. E. & Stunkard, A. J. (1965) Social Factors in Obesity. *J. Am. med. Ass.*, **192**, 97.

Hems, G. (1970) Epidemiological Characteristics of Breast Cancer in Middle and Late Age. *Br. J. Cancer*, **24**, 226.

Hems, G. & Stuart, A. (1975) Breast-cancer Rates in Populations of Single Women. *Br. J. Cancer*, **31**, 118.

Hotelling, H. (1940) The Selection of Variates for use in Prediction with some Comments on the General Problem of Nuisance Parameters. *Ann. Math. Stat.*, **11**, 271.

Kekwick, A. & Pawan, G. L. S. (1956) Calorie Intake in Relation to Body-weight Changes in the Obese. *Lancet*, ii, 155.

Keys, A., Anderson, J. T. & Grande, F. (1965) Serum Cholesterol Response to Changes in the Diet. *Metabolism*, **14**, 747.

Kumaoka, S., Abe, O., Utsunomiya, J., Bulbrook, R. D., Hayward, J. L. & Swain, M. C. (1973) Plasma Oestradiol and Urinary Oestrogen Metabolites in Normal Japanese and British Women. In *Host environment Interaction in the Etiology of Cancer in Man*, Ed. Doll, R., Vodopija, I. & Davis, W. Lyon: Int. Agency for Res. on Cancer.

Lamming, G. E. (1966) Nutrition and the Endocrine System. *Nutr. Abstr. Rev.*, **36**, 1.

McCance, R. A. & Widdowson, E. M. (1960) *The Composition of Foods Medical Research Council Special Report Series No.* 297. London: H.M.S.O.
MacMahon, B. M., Cole, P. & Brown, J. (1973) Etiology of Human Breast Cancer: a Review. *J. natn Cancer Inst.*, **50**, 21.
MacMahon, B., Cole, P., Brown, J. B., Aoki, K., Lin, T. M., Morgan, R. W. & Woo, N. (1974) Urine Oestrogen Profiles of Asian and South American Women. *Int. J. Cancer*, **14**, 161.
Marsden, R. A. (1965) The Management of Hypercalcaemia in Malignant Disease. *Med. J. Aust.*, **2**, 411.
Matthiessen, P. C. (1970) *Some Aspects of the Demographic Transition in Denmark.* Copenhagen: University.
Medina, D. (1974) Mammary Tumourgenesis in Chemical Carcinogen-treated Mice. II. Dependence on Hormonal Stimulation for Tumourgenesis. *J. natn Cancer Inst.*, **53**, 223.
Mirra, A. P., Cole, P. & MacMahon, B. (1971) Breast Cancer in an Area of High Parity: Sao Paulo, Brazil. *Cancer Res.*, **31**, 77.
National Bureau of Economic Research (1960) *Demographic and Economic Change in Developed Countries.* Princeton: University Press.
Phillips, R. L. (1975) Role of Life-style and Dietary Risks in Risk of Cancer among Seventh-Day Adventists. *Cancer Res.*, **35**, 3513.
Ravnihar, B., MacMahon, B. & Lindtner, J. (1971) Epidemiologic Features of Breast-cancer in Slovenia. *Eur. J. Cancer*, **7**, 295.
Segi, M. & Kurihara, M. (1966) *Cancer Mortality for Selected Sites in* 24 *Countries.* Sendai, Japan.
Segi, M. Kurihara, M. & Matsuyama, T. (1969) *Cancer Mortality for Selected Sites in* 24 *Countries. No.* 5 (1964–65). Sendai, Japan.
Segi, M. & Kurihara, M. (1972) *Cancer Mortality for Selected Sites in* 24 *Countries. No.* 6 (1966–67). Nagoya: Japan Cancer Society.
Segi, M. (1975) *Age-adjusted Death Rates for Selected Sites (A-classification) in* 43 *Countries in* 1970. Nagoya: Japan Cancer Society.
Segi, M. & Tomatsu, K. (1976) *Age-adjusted Death Rates for Selected Sites (A-classification) in* 43 *Countries in* 1971). Nagoya: Japan Cancer Society.
Snedecor, G. W. & Cochran, W. G. (1967). *Statistical Methods.* Iowa: State University Press.
Sommers, S. C. (1955) Endocrine Abnormalities in Women with Breast Cancer. *Lab. Invest.*, **4**, 160.
Stamler, J., Stamler, R. & Shekelle, R. B. (1970) Regional Differences in Prevalence, Incidence and Mortality from Athero-sclerotic Heart Disease. In: *Ischaemic Heart Disease.* Ed. De Haas, J. H., Hemker, H. C. & Snellen, H. A. Leiden: University Press.
United Nations (1950) *Demographic Yearbook.* New York.
United Nations (1969) *Demographic Yearbook.* New York.
United Nations (1975) *Demographic Yearbrook.* New York.
Valaoras, V. G., MacMahon, B., Trichopoulos, D. & Polychronopoulou, A. (1969) Lactation and Reproductive Histories of Breast-cancer Patients in Greater Athens. *Int. J. Cancer*, **4**, 350.
W.H.O. (1970) *Health Stat. Rep.*, **23**, 1177.
de Waard, F. & Baanders-van Halewijn, E. A. (1974) A Prospective Study in General Practice on Breast-cancer Risk in Post-menopausal Women. *Int. J. Cancer*, **14**, 153.
de Waard, F., de Laive, J. W. J. & Baanders-van Halewijn (1960) On the Bi-modal Age Distribution of Mammary Carcinoma. *Br. J. Cancer*, **14**, 437.
Williams, E. J. (1959) *Regression Analysis.* London: Wiley.
Wynder, E. L. (1968). Identification of Women at High Risk for Breast Cancer. *Cancer*, **21**, 1235.
Zubiran, S. & Gomez-Mont, F. (1953) Endocrine Disturbances in Chronic Human Malnutrition. *Vitams Horm.*, **11**, 97.